# Lipidomic profiling of the cerebrospinal fluid in moyamoya angiopathy patients

**DOI:** 10.1186/s13023-025-03782-5

**Published:** 2025-05-23

**Authors:** Antonella Potenza, Gemma Gorla, Tatiana Carrozzini, Giuliana Pollaci, Michele Dei Cas, Francesco Acerbi, Ignazio G. Vetrano, Paolo Ferroli, Isabella Canavero, Rita Paroni, Nicola Rifino, Anna Bersano, Laura Gatti

**Affiliations:** 1 Laboratory of Neurobiology and UCV, Neurology IX Unit, Fondazione IRCCS Istituto Neurologico Carlo Besta, 20133 Milan, Italy; 2 Department of Pharmacological and Biomolecular Sciences, Università degli Studi di Milano, 20122 Milan, Italy; 3 Clinical Biochemistry and Mass Spectrometry Lab, Department of Health Sciences, Università degli Studi di Milano, 20132 Milan, Italy; 4 Neurosurgical Unit, Fondazione IRCCS Istituto Neurologico Carlo Besta, 20133 Milan, Italy; 5 Department of Translational Research and New Technologies in Medicine and Surgery, University of Pisa, 56126 Pisa, Italy; 6 Neurosurgery Unit, Pisa University Hospital, 56126 Pisa, Italy; 7 Department of Biomedical Sciences for Health, Università degli Studi di Milano, 20122 Milan, Italy

**Keywords:** Moyamoya, CSF, Plasma, Lipidomics, Phospholipids, Sphingolipids, Phosphatidylcholines, Inflammation

## Abstract

**Background:**

Moyamoya angiopathy (MA) is a rare cerebrovascular disorder which can occur in both children and young adults, characterized by progressive occlusion of the intracranial carotid arteries, leading patients to ischemic and haemorrhagic strokes. Despite decades of research, the mechanisms underlying MA remain poorly clarified and current gaps in the understanding of pathogenesis have hampered the development of suitable preventive strategies and therapeutic options. Moreover, clinically approved biomarkers for MA patients’ stratification are missing. The unknown pathophysiology and the lack of reliable biomarkers prompted us to investigate cerebrospinal fluid (CSF) lipidome through state-of-the-art lipidomics.

**Methods:**

Intraoperative CSF from a subgroup of MA patients in comparison to age/sex matched controls (CTRL) was analysed through LC–MS/MS, by an untargeted lipidomic approach. Receiver operating characteristic (ROC) curve and simple linear regression analyses were performed for diagnostic use. We searched for simultaneously altered lipids in plasma and CSF of MA patients.

**Results:**

Overall, we observed a significant increase of sphingolipids (*p* < 0.05) and phospholipids (*p* < 0.05) in MA CSF. A partial least squares discriminant analysis clearly separated MA and CTRL by 64% on Principal Component 1. We identified lipid classes (n = 12) with a Variance Importance in Projection score ≥ 1.5, within those lipids highly correlated with MA (n = 70). A significant increase in acylcarnitines, sphingolipids (sphingomyelins and ceramides), phospholipids (lysophosphatidylcholines; phosphatidylcholines; phosphatidylethanolamines; ether-phosphatidylethanolamines; ether-phosphatidylcholines) and cholesterol esters was found by multivariate and univariate analyses. Monoacylglycerols were the only lipid class displaying a markedly significant (*p* < 0.001) decrease in CSF of MA patients as compared to CTRL subjects. The ROC curve and simple linear regression analysis identified 10 out of 12 lipid classes as reliable MA biomarkers, mainly dealing with phospholipids. We then compared current and previous data on plasma lipidomic profile. The discriminant analysis returned n = 175 (in plasma) and n = 70 (in CSF) simultaneously altered lipids respectively, and phosphatidylcholines (n = 10) resulted as commonly decreased in plasma and increased in CSF.

**Conclusions:**

Our findings highlighted a strong pro-inflammatory environment in MA CSF. These preliminary hallmarks could be helpful to decipher the complex MA pathogenesis, by supplying candidate biomarkers for patient stratification.

**Supplementary Information:**

The online version contains supplementary material available at 10.1186/s13023-025-03782-5.

## Background

Moyamoya angiopathy (MA) is an uncommon non-atherosclerotic cerebrovascular disease, characterized by progressive stenosis of the terminal internal carotid artery and its proximal branches, and formation of hazy collateral vessels at the base of the brain. The main clinical manifestations of MA are recurrent ischemic or haemorrhagic strokes and cognitive impairment, which can occur in both children and young adults [1–3]. Despite decades of research, the etiology and pathophysiological mechanisms underlying MA remain poorly clarified. Previous studies have highlighted the potential involvement of inflammation as a cause or progressive factor of MA, although the role of inflammation has not been rigorously explored [4, 5]. Inflammatory processes have been suggested to instigate or exacerbate this rare condition, particularly in pediatric patients [6]. MA is also emerging as an immune-related angiopathy, since an increasing number of reports describes the development of MA associated with infection or autoimmune disorders [7, 8]. Several studies have identified a variety of autoimmune diseases associated to MA [9, 10]. Besides, recent reports suggested that a more aggressive MA course is associated with the presence of thyroid autoantibodies [11]. Together, this growing body of molecular and clinical evidence points towards immune-related responses as second hits to trigger MA onset and may hint at therapeutic opportunities. 

Current gaps in the understanding of disease pathogenesis have hampered the development of suitable preventive strategies and therapeutic options. Surgical revascularization, including superficial temporal artery (STA) to middle cerebral artery bypass (MCA), has been proven as an effective treatment to prevent subsequent strokes of MA patients [12]. The prevalence of MA varies widely among ethnic groups, thus genetic factors have been implicated in disease development [13, 14]. Specifically, a high prevalence of MA patients has been reported among the descendants belonging to East Asian countries, of whom the three most common are Japan, Korea and China. The estimated prevalence of MA in Japan is 10.5/100,000 individuals and the incidence rate is 0.94/100,000; conversely, the incidence rate is almost 10-fold lower accounting for 0.09/100,000 individuals in Western regions, including North America [15]. Additionally, the incidence of MA in female subjects has been reported to be higher than that in males. Furthermore, a bimodal age distribution has been described is MA patients, referring to a highest peak observed at 45–49 years and a smaller peak observed at 5–9 years [16]. The main susceptibility gene variant identified in East Asian patients encodes for the ubiquitin-ligase Ring Finger Protein 213 (RNF213) [17]. So far, no animal or cellular preclinical model accurately recapitulates the pathological changes observed in vascular intimal hyperplasia of MA patients [18]. Moreover, there are no clinically approved biomarkers for distinguishing among the different subtypes of MA.

The previous investigations aimed at biomarker discovery mainly addressed peripheral blood, which may not reflect the real pathological changes of MA cerebral vessels. Since the challenging sampling of cerebral artery specimens for transcriptomic studies, other ultrasensitive techniques were recently carried out for molecular profiling of circulating biomarkers [19–21]. In this regard, multi-omic approaches may turn out to be crucial in patients’ risk stratification and precision medicine, allowing for the selection and customization of medical treatment based on individual signatures [22]. Despite numerous findings in the proteomics and metabolomics field [23–27], very little is known about changes in the lipid composition in patients’ circulating fluids, although it has been already reported that RNF213 is involved in lipid metabolism [28]. Lipids are a significant part of the neural molecular environment, constituting approximately 50% of the dry weight of the brain [29]. They are essential for proper brain function, being required for axon growth and potentiation, membrane trafficking, myelin formation, synaptogenesis and accounting for critical components of cell membranes [30]. It is not surprising that changes that affect brain lipid metabolism and/or its delicate vascular network, have profound implications in tissue homeostasis and have been implicated as underlying mechanisms for many cerebrovascular diseases. The alterations in the MA vasculature induced by ischemic insult and oxygen deprivation can cause the loss of astrocytes and microglia, reduce neuronal function and increase neuroinflammation. These changes may be coupled with intense lipid remodeling in CSF, and affect also cholesterol metabolism, which is essential for neuronal growth and survival since they need cholesterol to repair and build more membranes.

Recently, a lipidomic analysis of cerebral arteries in MA and intracranial atherosclerotic disease patients identified distinct lipid species for distinguishing between the two conditions [31]. Our previous gene expression analyses of MA pathological cerebral vessels also underlined the relevance of specific biomarkers for patient’s stratification, as well as a novel potential therapeutic target for MA care [32]. However, the poor availability and difficulty in collecting arterial tissue specimens prompted us to search for valuable biomarkers in circulating fluids, mainly plasma and CSF. We previously performed an untargeted lipidomic analysis showing a cumulative depletion of lipid asset in plasma of MA as compared to healthy donors [33]. These promising but preliminary results encouraged us to investigate cerebrospinal fluid (CSF) as an alternative biomarker source. The identification of changes in metabolic profiles, providing valuable biomarkers and shedding light on mechanisms of diseases could have a crucial role in patients’ risk stratification and precision medicine, allowing for the selection and customization of medical treatment based on individual metabolic profiles. In this pilot study, we aimed to elucidate the MA CSF lipidome, by identifying altered lipids in a representative sample of a typical Caucasian MA population. We consider that a better understanding of specific lipid changes and their eventual correlation with clinical outcomes could help to guide patients’ stratification approaches, with a possible impact also in the prevention of MA evolution.

## Methods

### Cohort selection and sampling

This was an observational study conducted on MA patients, diagnosed following the literature criteria [34]. The full methodology of the study has been previously reported [35]. From the original population of 160 patients, consecutively enrolled between November 2014 and December 2022 at the Neurology IX Unit of the Fondazione IRCCS Istituto Neurologico “C. Besta” (Italy, Milan), a subgroup of five adult Caucasian patients was selected, exclusively based on CSF availability and demographic/clinical features typical of MA cohort. CSF samples were obtained after the neurological insult that determined the STA-MCA neurosurgical bypass, according to European Stroke Organisation (ESO) Guidelines on moyamoya angiopathy [36]. The study design was approved by the Ethics Committee of the Fondazione IRCCS Istituto Neurologico “C. Besta” of Milan (GEN-O-MA clinical study, report no. 12, 10/01/2014). Informed written consent for study participation and sample collection from all patients and controls were mandatory for study inclusion. Demographic and clinical characteristics, including cerebrovascular risk factors, were collected. MA was classified into bilateral or unilateral types as observed on conventional angiography. Diagnosis of ischemic or haemorrhagic stroke was confirmed by computerized tomography scan and magnetic resonance imaging. The MA severity was assessed by Suzuki and modified Rankin scale according to standard clinical practice and international guidelines [35–37]. A population of five adult Caucasian subjects admitted to our Institution and suffering from MA-unrelated hemodynamic insufficiency, secondary to arterial stenosis due to atherosclerotic diseases or due to isolated intracranial aneurysms, was collected as control group (CTRL). Control CSF was sampled during surgical procedure, in patients undergoing STA-MCA bypass for hemodynamic cerebrovascular insufficiency or a craniotomy to clip intracranial aneurysm in the anterior circulation, mostly MCA or anterior communicating artery. No further procedures apart from the surgical treatment were necessary to obtain CSF. A multidisciplinary team discussed surgical indications in all the patients, both MA and CTRL. CSF was collected from MA patients and CTRL subjects from cisternal spaces, mainly sylvian fissure, after arachnoid incision, in compliance with ethical regulations of ESO Guidelines on moyamoya angiopathy [36]. After collection, samples were briefly centrifuged to remove any cellular debris and stored in aliquots at − 80 °C. Twenty-four milliliters of peripheral blood were collected in tubes containing EDTA (Vacuette®, Preanalitica s.r.l., Caravaggio, Italy). Blood samples were analyzed for levels of triglycerides (TG), total cholesterol (CHOL) and high-density lipoprotein (HDL) CHOL by standard clinical laboratory methods; low-density lipoprotein (LDL) CHOL concentrations were estimated as CHOL—(HDL + TG/5). After 10 min at 200 g centrifugation, plasma was stored in aliquots at − 80 °C.

### Chemicals and reagents

The chemicals acetonitrile, 2-propanol, methanol, chloroform, formic acid, ammonium acetate, ammonium formate and dibutylhydroxytoluene (BHT) were purchased from Sigma-Aldrich (St. Louis, MO, USA). All aqueous solutions were prepared using purified water at a Milli-Q grade (Burlington, MA, USA).

### Untargeted lipidomics

CSF and plasma were diluted with water (1:2 CSF, 1:4 plasma v/v) and lipids were extracted by a mixture of methanol/chloroform (850 µL, 2:1, v/v). The samples were extracted with an oscillator thermo-mixer (60 min, 1000 rpm, 4 °C). After centrifugation (25 min at 13400 rpm), the organic phase was evaporated under a stream of nitrogen. The residues were dissolved in 100 µL of methanol + 0.1% BHT and withdrawn in a glass vial. Extraction and analysis performances were checked by the use of a preconstituted internal standards mix (Equisplash Lipidomix, 150 ng per sample, Avanti Polar, Alabaster, AL, USA). The extracts were analysed by LC-MS/MS consisting of a Shimadzu UPLC coupled with a Triple TOF 6600 Sciex (Concord, ON, CA) [33]. All samples were analyzed in duplicate in positive and negative electrospray ionization. Spectra were contemporarily acquired by full-mass scan from 200 to 1500 m/z and top-20 data-dependent acquisition from 50 to 1500 m/z. Declustering potential was fixed to 50 eV, and the collision energy was 35 ± 15 eV. The chromatographic separation was reached on a reverse-phase Acquity CSH C18 column 1.7 μm, 2.1 × 100 mm (Waters, Franklin, MA, USA) by a gradient between (A) water/acetonitrile (60:40) and (B) 2-propanol/acetonitrile (90:10), both containing 10 mM ammonium acetate and 0.1% of formic acid.

### LC-HR-MS data processing

The spectra deconvolution, peak alignment, and sample normalization were attained using MS-DIAL [38]. MS and MS/MS tolerance for peak profile was set to 0.01 and 0.05 Da, respectively. Identification was achieved matching spectra with LipidBlast database or in-house built mass spectral library. Intensities of analytes were normalized by Lowless algorithm and those with a CV% superior to 30% in the QC pool sample were excluded. The lipid classes considered were acylcarnitines (ACar), cholesterol (chol), cholesterol esters (CE), monoacylglycerols (MAG), diacylglycerols (DAG), triacylglycerols (TAG); dihydroceramides (DHCer), ceramides (Cer), hexosylceramides (HexCer), lactosylceramides (LacCer), globotriaosylceramide (Gb3), sulfatides (SULF), gangliosides (GM3), sphingomyelins (SM), lysophosphatidylcholines (LPC), lysophosphatidylethanolamines (LPE), phosphatidylcholines (PC), phosphatidylethanolamines (PE), phosphatidylinositoles (PI) and plasmalogens – that are ether linked phosphatidylcholines (EtherPC) and vinyl linked phosphatidylethanolamines (EtherPE). The lipids are indicated throughout the paper by their total number of carbon atoms and degree of unsaturation (i.e. PC 40:2) or eventually specifying the acyl chains detected (i.e. PC 18:0/18:1). The study raw data supporting lipidomic reported results can be found in the public repository MetaboLights [39].

### Statistical analyses

Graphs and statistical analyses were prepared with GraphPad Prism 8.0, and with MetaboAnalyst5.0 [40]. Statistical significance was evaluated by comparing ranks with non-parametric Mann-Whitney U test and Fisher’s exact test. Multivariate analysis partial least squares discriminant analysis (PLS-DA) was performed to increase the group separation and investigate the variables with a high Variance Importance in Projection score (VIP > 1.5). *P* <0.05 was considered statistically significant. *P* values are schematized as follows: * < 0.05; ** < 0.01; *** < 0.001. Data are shown as mean ± SD. To explore factors affecting the lipid composition within MA patients, receiver operating characteristic (ROC) curves and simple linear regression model were used. Sensitivity and specificity were defined to estimate the performances of the candidate lipid to discriminate between MA patients and CTRL. The authors were blinded to the experimental protocol while performing the experiments and the statistical calculations.

## Results and discussion

### Demographic characteristics of the study cohorts

According to the GEN-O-MA project and ESO Guidelines [35, 36], a small but representative subgroup of subjects was enrolled for the present study, since untargeted lipidomics requires a very homogeneous group that would allow identifying any potetial difference in lipidome profiles between MA patients and CTRL subjects. Specifically, we included five MA Caucasian patients, based on CSF availability and demographic/clinical features typical of our MA cohort (Caucasian, females, mean age of 47 years, bilateral MA presentation) [33, 41]. Of note, these characteristics are precisely representative of the whole MA population in Western countries, which is a relevant aspect considering the rarity of the disease [42]. We summarized MA patient information in Table 1, by reporting the mean age (± SD); Sex (female %); cerebrovascular disease (CVD) type (hemorrhagic stroke, ischemic stroke, transient ischemic attack TIA); National Institutes of Health Stroke Scale (0–5; 6–10); MA presentation (Unilateral or Bilateral); Suzuki grading (I–III; IV–VI); modified Rankin Scale (0–1; 2–3). The patients (100% females) had a mean age of 45.56 ± 4.2 years (range 38.8–49.6). The disease presented with a haemorrhagic stroke in 40% of patients, an ischemic event in 40% and a TIA in 20% of the cases. Five control patients (100 % females; mean age of 66.2 years, range 44.0–76.0) underwent conventional catheter digital subtraction angiography and morphological imaging by MRI, likewise MA patients. Computerized tomography scan and MRI showed an ischemic stroke in three controls out of five (corresponding to a percentage of 60%) and a TIA in one control subject out of five (corresponding to a percentage of 20%), representing patients with hemodynamic cerebrovascular impairment, deeply distinct and unrelated from MA. The hemorrhagic phenotype was reported in one control patient out of five (corresponding to a percentage of 20%), who exhibited an intracranial aneurysm with previous small bleeding (Table 1).

**Table 1:** Participant characteristics^a^

		MA	CTRL	*P* value
N		5	5	
Mean age (y ± SD)		45.56 ± 4.2	66.2 ± 12.77	0.095 (ns)
Sex (% F)		100%	100%	1 (ns)
CVD type	HS	40%	20%	1 (ns)
	IS	40%	60%	1 (ns)
	TIA	20%	20%	1 (ns)
NIHSS	0–5	80%	na	#
	6–10	20%	na	#
MA presentation	U	0%	na	#
	B	100%	na	#
Suzuki grading	I–III	40%	na	#
	IV–VI	60%	na	#
mRS	0–1	100%	100%	1 (ns)
	2–3	0	0	#
CHOL (mg/dl)		168.6	217.7	0.571 (ns)
TG (mg/dl)		102.2	176.3	0.589 (ns)
HDL (mg/dl)		40	35	0.467 (ns)
LDL (mg/dl)		92.25	92	0.999 (ns)

^a^Demographic, clinical, and neuroradiological features of MA patients and control subjects. Statistical significance was evaluated by comparing ranks with Mann–Whitney U test or Fisher’s exact test. B, bilateral; CHOL, total cholesterol; CTRL, controls; CVD, cerebrovascular disease; F, female; HDL, high-density lipoprotein—cholesterol; HS, haemorrhagic stroke; IS, ischemic stroke; LDL, low-density lipoprotein—cholesterol; MA, moyamoya angiopathy; mRS, modified rankin scale; N, number of subjects; na, not available; NIHSS, national institutes of health stroke scale; ns, not significant; TIA, transient ischemic attack; TG, triglycerides; U, unilateral; y, years

No statistical difference has been evidenced when comparing peripheral blood lipid levels determined by standard clinical laboratory methods, in MA patients and CTRL (CHOL; HDL; LDL; TG; Table 1).

### Increase of the overall lipid content in CSF of MA patients.

**Fig. 1:**
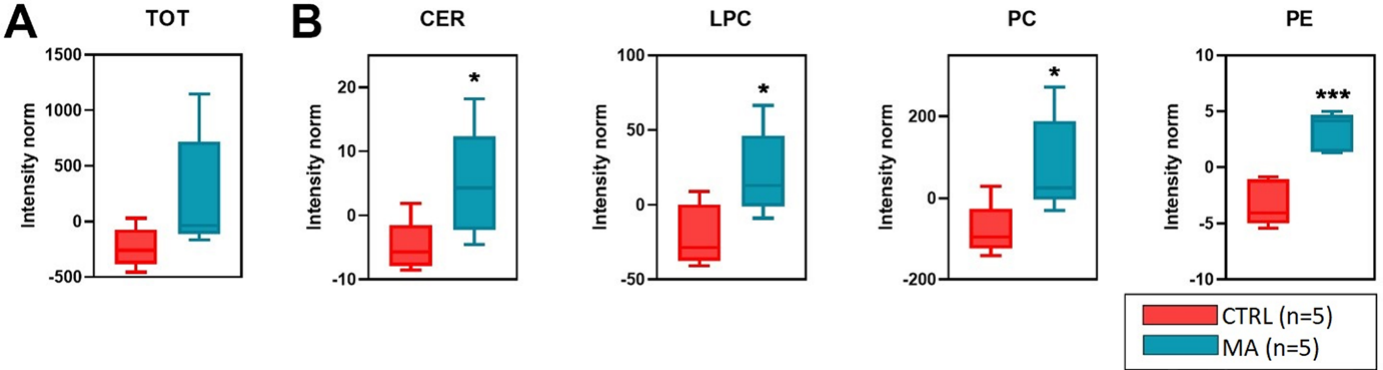
Overall CSF lipid content. **A** Total CSF lipid content in MA patients and in CTRL subjects with unrelated cerebrovascular diseases **B** Lipid classes significantly increased in CSF of MA patients. Statistical significance is evaluated by comparing ranks with Mann–Whitney U test. *P* values are schematized as follows: * < 0.05; *** < 0.001

A total of n = 623 different lipids were identified and quantified in CSF from MA and CTRL populations, by an untargeted lipidomic approach. The overall CSF lipid content of MA patients was higher in comparison to CTRL, although not statistically significant (Fig. 1A).

Among the most altered lipid categories, we found the sphingolipids (Ceramides, Cer) and phospholipids (i.e., LPC; PC; PE). In particular, we observed significant increases for Cer (*p *< 0.05), LPC (*p *< 0.05), PC (*p *< 0.05) and PE (*p *< 0.001; Fig. 1B) classes. A list of the main lipid classes emerging from our analysis was shown (Table 2). Of note, although characterized by a different time lapse (25 ± 13 months) between disease onset and CSF sampling during the neurosurgical procedure, the lipidome profile of our MA cohort is quite homogeneous (see Additional File 1). Moreover, when we performed appropriate statistical analyses (non-parametric Mann-Whitney U test), to evaluate the profile of specific CSF lipid classes/species in MA patients in relation to CVD type (HS vs IS/TIA), Suzuki grading (I–III vs. IV–VI) and NIHSS (0–5 vs. 6–10), we did not find any statistically significant difference between the analysed subgroups (see Additional Files 2, 3, 4).

**Table 2:** Nomenclature of CSF lipid classes/species* emerging from lipidomics

ACar	Acylcarnitines	MAG	Monoacylglycerols
Cer 18:0	Ceramide 18:0	LPC	Lysophosphatidylcholines
CE 22:4	Cholesterol ester 22:4	PC	Phosphatidylcholines
DAG	Diacylglycerols	PE	Phosphatidylethanolamines
Ether PC	Phosphatidylcholine Ethers	SM	Sphingomyelins
Ether PE	Phosphatidylethanolamine Ethers	TAG	Triacylglycerols

^*^Cer 18:0 and CE 22:4 were included in Table because were found as single lipid altered species of their respective lipid classes

Ceramides, beyond being an essential constituent of cytomembrane, play a key role as signaling mediators in many cell processes and stress responses. Interestingly, a marked relation has been documented between ceramides and cerebrovascular diseases [43]. PC act as principal components of cell membranes, playing a role in cell signalling and ameliorating neuronal damage under inflammatory stress, by re-establishing plasticity of neural stem cells [44, 45]. LPC, as well as PC, are bioactive compounds associated with oxidative stress and inflammatory processes, with a potential effect in preventing ischemic injury or neuronal death in patients with stroke [46, 47]. Interestingly, an untargeted metabolomic approach recently carried out has been able to identify a distinct metabolite expression, mostly dealing with LPC, in serum samples from MA patients when compared to controls [27]. Specifically, LPC 16:1-2 expression was found as significantly lower in ischaemic MA patients than in healthy controls. The LPC-enriched CSF lipid content reported in the present study might counterbalance the corresponding LPC depletion identified in plasma samples of the same MA cohort [33].

### Differential lipid analysis

**Fig. 2:**
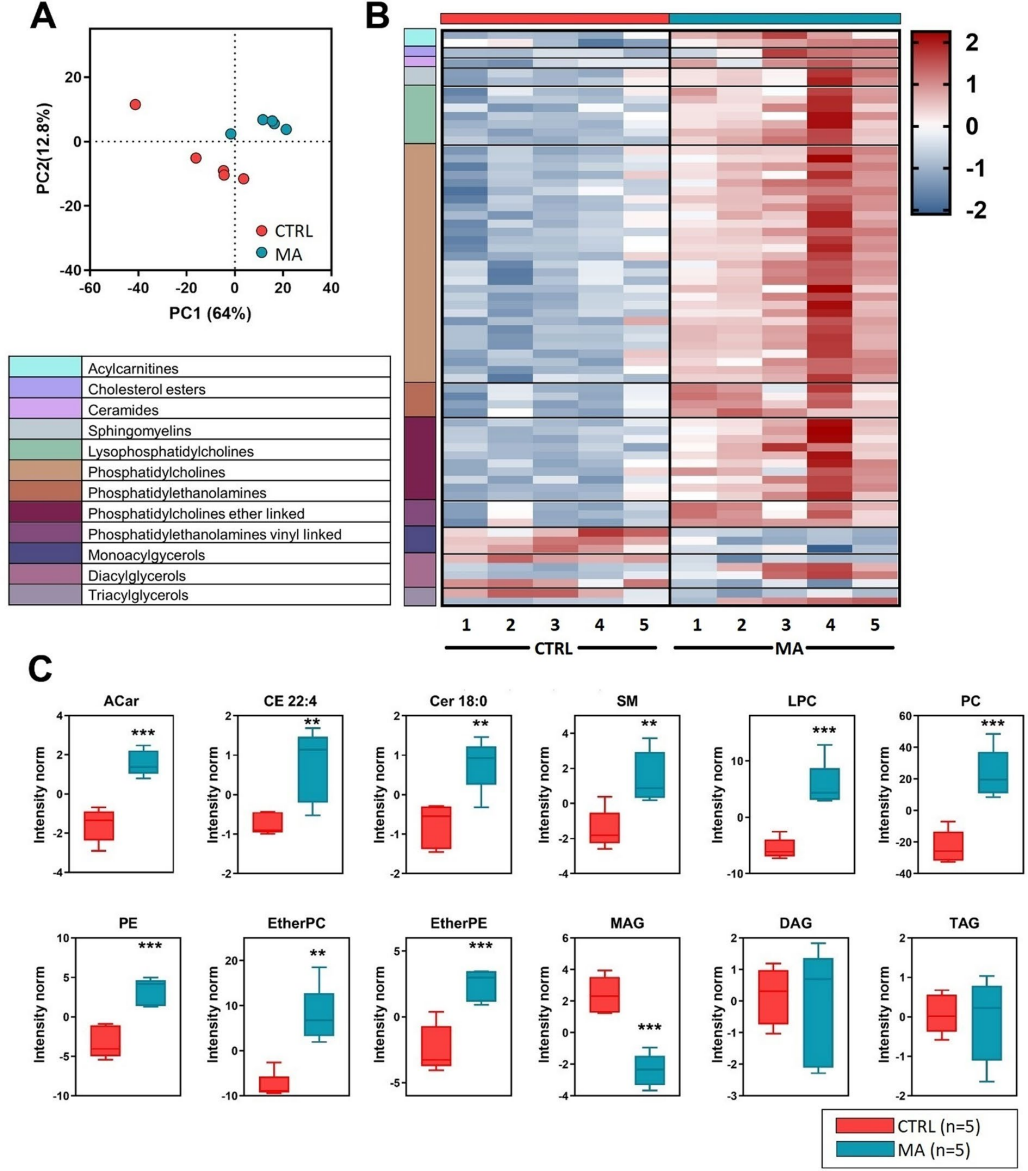
CSF untargeted lipidomics. CSF lipid alteration in MA patients (n = 5) in comparison to age and sex matched controls with unrelated cerebrovascular diseases (CTRL, n = 5). **A** Discriminant analysis (score plot) of the lipidome in MA and CTRL subjects. The axes are ranked according to their importance in the group discrimination. In the x-axis, component 1 (PC1, 64%) represents the maximum of the separation that can be reached within these clusters and variables, whereas, in the y-axis, component 2 (PC2, 12.8%) represents the direction that contains the most remaining variance. **B** Heatmap of the top 70 significantly altered lipid species highly correlated with the disease, chosen within those with a Variance Importance in Projection (VIP) score > 1.5, and ordered by lipid classes, coded by different colors. Each horizontal row represents a molecular lipid; each vertical column represents a sample. The concentrations were autoscaled and log- transformed for visualization. The color code bar indicates the log of the fold change of the mean concentration for a given lipid. The color-scale differentiates values as high (red), average (white) and low (blue). **C** VIP lipid classes concentrations in MA and CTRL subjects. Boxplots represent the trends in lipid class concentrations in MA and CTRL patients, except for Cer 18:0 and CE 22:4 which are the only VIP lipids found in their classes and therefore the boxes represent the trend of these single species. The boxes represent data obtained in the range 25th-75th percentile; the line across the boxes indicates the median value; the lines above and below the boxes indicate extreme values (10th or 90th percentile). Outliers are displayed as separate points. Statistical significance is evaluated by comparing ranks with Mann–Whitney U test. *P* values are schematized as follows: ** < 0.01; *** < 0.001

A PLS-DA was performed to identify deregulated lipids associated with MA (Fig. 2A). This analysis clearly separated MA patients and CTRL subjects by 64% on Principal Component 1 (PC1). The PC1 represents the new dimension in which the initial variables are compressed and depicts the maximum of the separation that can be reached within these clusters and variables. The Variance Importance in Projection (VIP) scores derived from PLS-DA were used for ranking the discriminating features, taking a cut-off value ≥ 1.5. Those lipid species (*n* = 70) with a VIP ≥ 1.5 that significantly contributed to the separation of MA patients and CTRL are visualized by a heatmap and ordered according to their lipid classes (Fig. 2B). Fig. 2C shows a univariate analysis with the twelve lipid classes displaying significant modulations due to the disease. Features exhibiting VIP ≥ 1.5 and variance *p*-value ≤ 0.05 were selected as significantly differentially abundant lipids. Of note, Cer 18:0 and CE 22:4 are the only VIP lipids found in their corresponding lipid classes (i.e., sphingolipids and neutral lipids), and therefore the boxes represent the trend of these single species.

The high sphingolipid/phospholipid content as well as the finding of pro-inflammatory lipid classes were confirmed by PCA. By evaluating both multivariate and univariate analyses, it has been found a particular increase in many phospholipids (PC, PE, LPC, Ether PC and Ether PE) and in Acylcarnitines (ACar; *p *< 0.001). The increased levels of ACar among acute ischemic stroke patients indicate a major energy requirement due to a negative feedback from the brain hypoxic state [48]. ACar are also related to the recovery of stroke [49], and carnitines are associated with ischemic strokes [50]. Moreover, a previous targeted metabolomic approach suggested a biomarker pattern including ACar for differential diagnosis of acute ischemic stroke [51]. Higher plasma ACar levels were observed in individuals with an increased cardiovascular disease risk, as shown by a greater mitochondrial production of reactive oxygen species, which increase vascular inflammation [52]. Ether-PC and ether-PE contain high amounts of arachidonic acid required for the biosynthesis of eicosanoids, bioactive lipids with a great role in inflammation. In hypertensive patients, a deficiency in plasma ether lipids has been demonstrated, specifically in ether-PC and ether-PE [53]. Moreover, an accelerated PC turnover in macrophages promotes adipose tissue inflammation in obesity [54]. Additionally, the role of LPC in promoting the release of inflammatory factors has been described in a wide range of diseases [55], thus raising the hypothesis that LPC-targeting might be a valuable therapeutic approach for inflammation-related diseases, including MA. The observed Cer 18:0 increase is a worse condition in several neurological diseases, due to proinflammatory and neurodegenerative functions [56]. Increased plasma levels of Cer (18:0) and SM have been implicated in insulin resistance, whereas inflammation may contribute to its long-term maintenance, especially in models of obesity-induced insulin resistance [57]. SM is reported to be a biomarker of demyelination [58], which is coherent with the occurrence of a myelin damage, observed in the white matter of MA patients [59]. The impact of sphingolipids is evident in acute lung injury, or chronic lung pathologies such as cystic fibrosis and chronic obstructive pulmonary disease, where inflammation has a major role [60]. CE in the human brain are sequestered as lipid droplets, primarily used for the transport and storage of CHOL [61], a process occurring allosterically in response to surplus CHOL within the cell [62]. CE are increased in several neurological disorders such as multiple sclerosis, Alzheimer’s disease, brain injury, Huntington’s disease and stroke [63, 64]. Recently, a shotgun lipidomic analysis reported that CHOL was inefficiently converted to CE in the blood of cardiovascular disease patients [65]. Interestingly, endogenously generated lipid mediators (branched FA esters of hydroxy FA) promote the resolution of inflammation and attenuate the microvascular and macrovascular complications of obesity and diabetes mellitus in chronic kidney disease [66]. This evidence highlights novel opportunities for potential therapeutic intervention through the targeting of pro-resolution, rather than anti-inflammatory pathways.

MAG was the only lipid class displaying a markedly significant (*p* < 0.001) decrease in CSF of MA patients as compared to CTRL subjects (Fig. 2C). MAG is a neutral lipid that is degraded to glycerol and fatty acids by MAG-lipase (MAGL), a proinflammatory enzyme with highest expression in brain [67, 68], where it constitutes the major source of arachidonic acid and proinflammatory prostaglandins [69]. The marked decrease of MAG in MA CSF might result in induction of pro-inflammatory signals. Interestingly, MAGL inhibition protects against inflammation and lesions induced by ischaemia/reperfusion injury [70]. Accordingly, inhibition of MAGL has emerged as an anti-inflammatory and protective option in several experimental models of chronic inflammatory diseases, by a mechanism that relies on a shift of lipid metabolism in inflammatory cells [67].

### ROC and simple linear regression analysis of lipid biomarkers

**Fig. 3:**
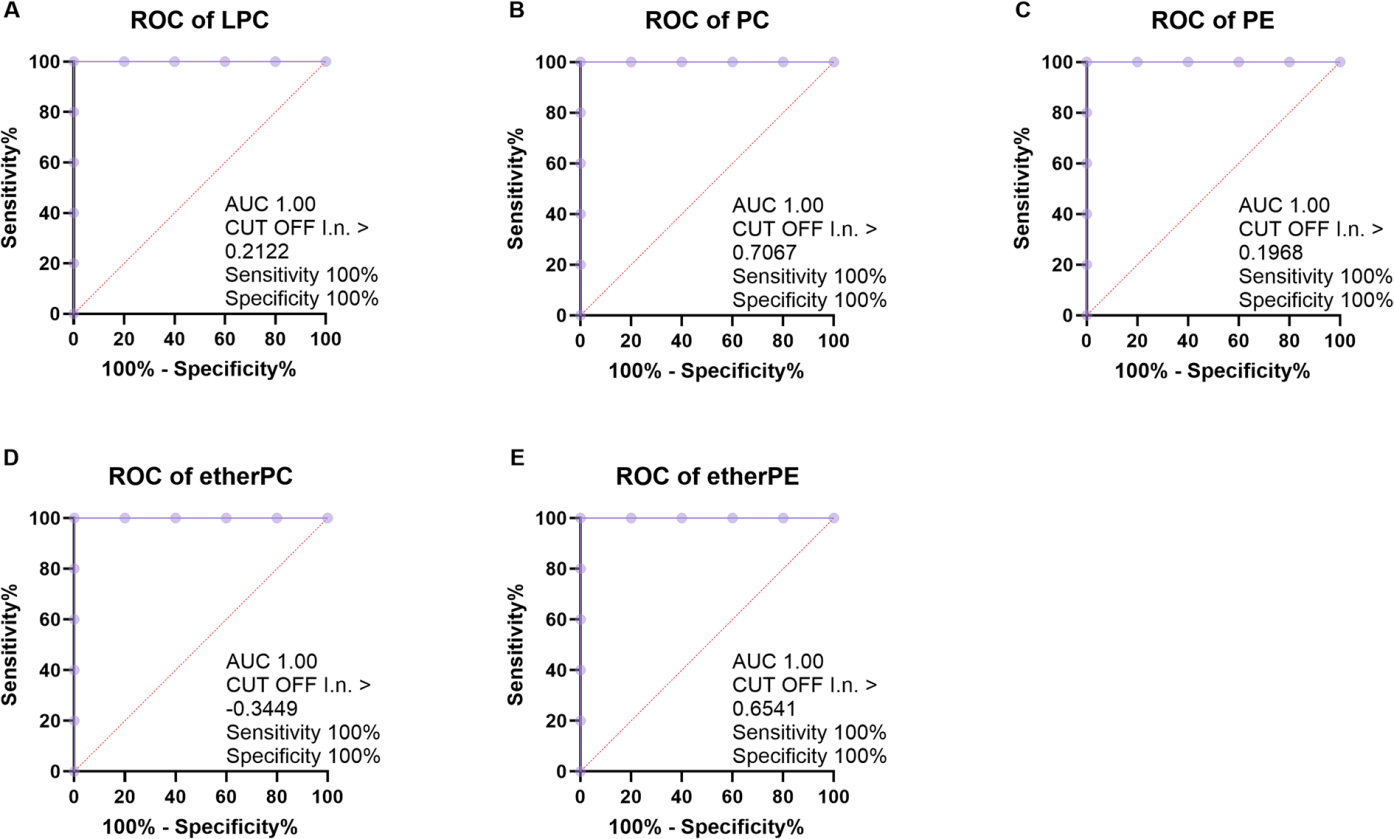
ROC analysis of phospholipid biomarker candidates. The performance of five lipid classes belonging to the phospholipid category (**A**–**E**), showing significant alterations in CSF (i.e., with VIP ≥ 1.5), for discriminating MA patients from CTRL subjects was evaluated by the area under the ROC curve (AUC) and by the determination of specificity (*percentage of true negatives*) and sensitivity (*percentage of true positive).* The nomenclature of lipid classes was reported above (see Table 2)

In order to determine whether these candidate CSF biomarkers could have any clinical utility as potential patient selection markers, we performed ROC curve and simple linear regression analysis for prospective follow-up and for diagnostic use. Twelve lipid classes showing significant alterations in CSF (i.e., with VIP ≥ 1.5) between MA and CTRL subjects were selected for individual ROC analysis. According to ROC analysis, 10 out of 12 classes proved to be candidate diagnostic biomarker, since they displayed high sensitivity and specificity performances (Table 3). All these 10 lipid classes presented area under the ROC curve (AUC) values higher than 0.9, and 7 out of 10 classes showed a perfect performance, by exhibiting an AUC = 1. Specifically, 5 out of 9 lipid classes showing a significant increase in MA patients belonged to the category of phospholipids (Table 3 and Fig. 3).

**Table 3:** ROC analysis of lipid biomarkers^a^

Lipid class	Trend	AUC	*P* value	CI
ACar	**↑**	1	0.009	1.000–1.000
LPC	**↑**	1	0.009	1.000–1.000
PC	**↑**	1	0.009	1.000–1.000
PE	**↑**	1	0.009	1.000–1.000
EtherPC	**↑**	1	0.009	1.000–1.000
EtherPE	**↑**	1	0.009	1.000–1.000
MAG	**↓**	1	0.009	1.000–1.000
SM	**↑**	0.96	0.016	0.843–1.000
CE 22:4	**↑**	0.92	0.028	0.739–1.000
Cer 18:0	**↑**	0.93	0.028	0.739–1.000
DAG	**↑**	0.52	0.917	0.122–0.918
TAG	**↑**	0.52	0.917	0.135–0.905

^a^Main lipid classes selected for individual receiver operating characteristic (ROC) curve analysis. The trend described by the analysis has been defined as enhanced (**↑**) or decreased (**↓**). P values and confidence interval (CI) values are reported. AUC, area under the ROC curve. Cer 18:0 and CE 22:4 are the only VIP lipids found in their classes and therefore they were reported as single species. The nomenclature of lipid classes/species was reported above (see Table 2)

**Fig. 4:**
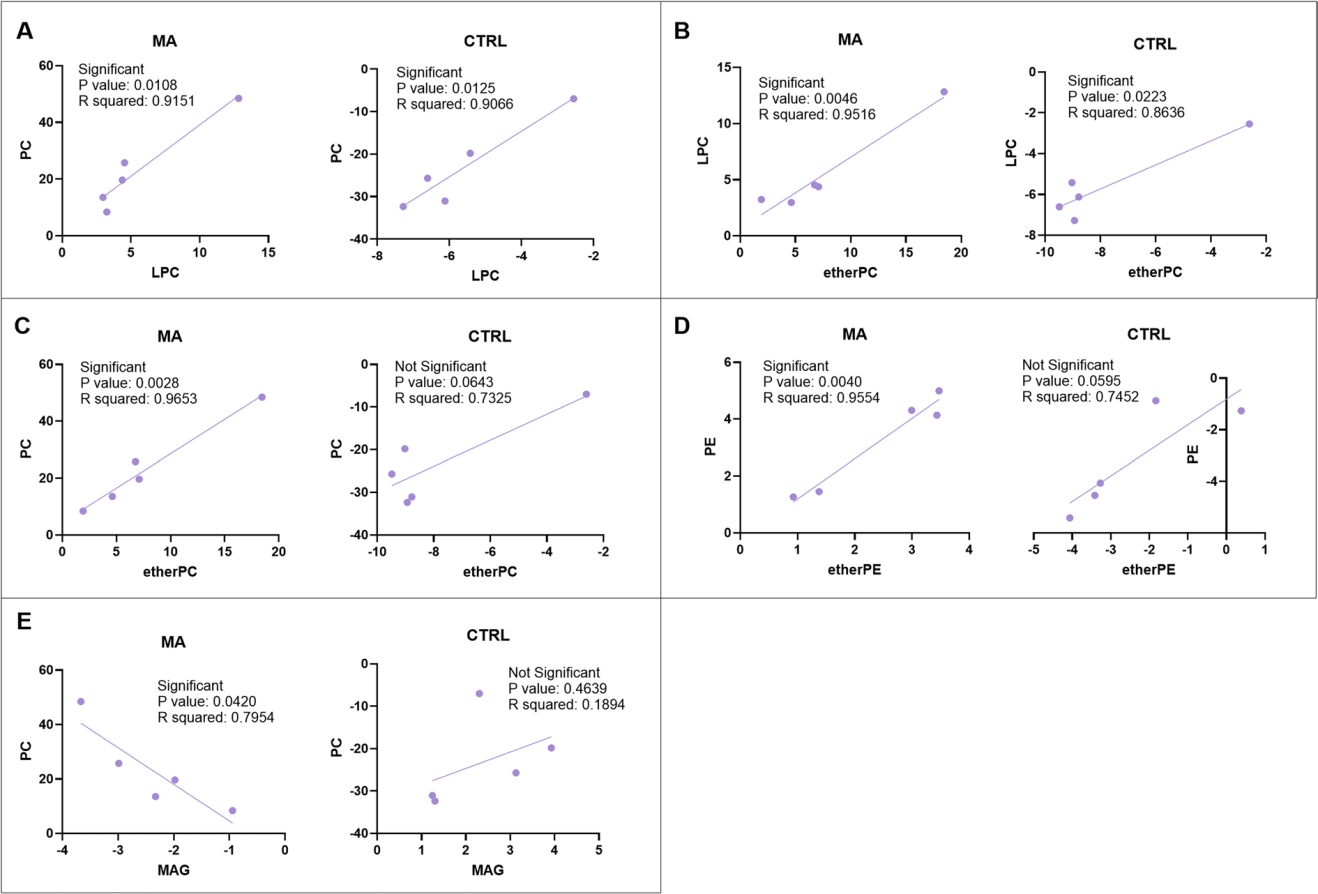
Simple linear regression model. Statistically significant simple linear regression analyses of selected lipid classes that showed VIP ≥ 1.5 in CSF of MA patients as compared to CTRL subjects; **A** PC/LPC; **B** LPC/ether PC; **C** PC/ether PC; **D** PE/ether PE; **E** PC/MAG. The nomenclature of lipid classes was reported above (see Table 2)

A simple linear regression model assayed the five phospholipid classes showing VIP ≥ 1.5 and exhibiting good performance after ROC analyses, to establish the reciprocal relationships among them (Fig. 4). Specifically, the expression levels of PC/LPC (Fig. 4A) and LPC/ether PC (Fig. 4B) increased proportionally in CSF of both MA patients and CTRL subjects. On the contrary, the relationships between PC/ether PC (Fig. 4C) and PE/ether PE (Fig. 4D) are directly proportional in CSF of MA patients but not in CTRL subjects. Other tested lipid classes showing linear correlation are shown in Additional File 5.

As we reported above, MAG was the only lipid class displaying a markedly significant (*p* < 0.001) decrease in CSF of MA patients as compared to CTRL subjects (Fig. 2C). Interestingly, PC/MAG (Fig. 4E) were correlated by an inverse proportionality only in MA patients’ CSF. Overall, we can hypothesize that the peculiar and significant correlation between PC increase and MAG decrease in patient’s CSF (Fig. 4E) could be suggestive of a MA-associated inflammatory environment.

### Discovery of lipids simultaneously altered in plasma and CSF of MA

By comparing current and previous data on plasma lipidomic profile of the same population of MA patients, we found that in CTRL subjects the overall CSF lipid content was 3% of the overall plasma lipid content [33]. Interestingly, in MA patients the CSF lipid content was 14% of the overall plasma lipid content, possibly due to the reported increase of Cer, LPC, PC and PE in MA CSF. Besides, a cumulative depletion of the plasma lipid asset was previously found in MA patients [33]. Conversely, the current lipidomic analyses depicted an overall lipid enrichment for MA CSF. Plasma and CSF lipid pools could be deeply different, since the first one is more prone to change, whereas the blood-brain barrier (BBB) isolates the central nervous system microenvironment from the peripheral vasculature under homeostasis [71, 72]. The relation between lipid decrease at the systemic and local levels still need to be established. It is therefore crucial to gain further insights into the lipid metabolism in inflammatory BBB disruption and the involved mechanisms. Upon the occurrence of strokes, vessels undergo several adverse stimuli that enhance their permeability and instability. CSF may better reflect the pathological changes observed in vascular intimal hyperplasia of MA patients, due to its proximity with the stenotic arteries and the compensatory network of “moyamoya” vessels. Using a myelin-sensitive MR imaging technique, Hara and colleagues found that myelin damage and axonal damage may exist in MA patients [59]. Accordingly, a BBB impairment was demonstrated in MA patients, mainly due to the typical fragile vascular system, to the Angiopoietin-2-related vascular plasticity and to endothelial dysfunction [32, 73].

**Fig. 5:**
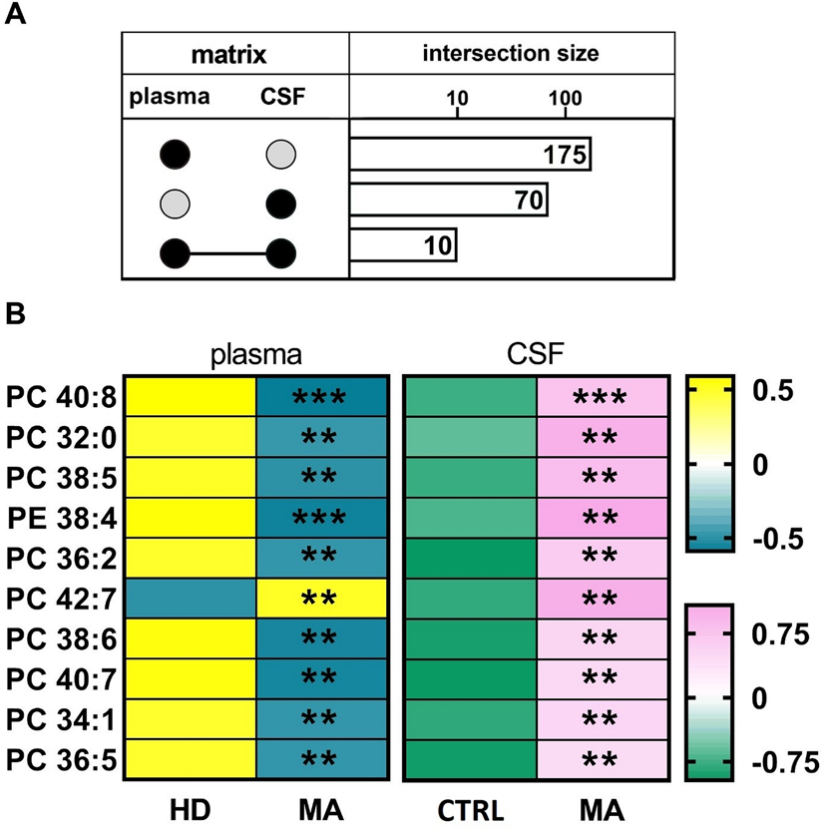
Common lipid features between plasma and CSF of MA. **A** Intersection size graph showing the number of lipids altered in both plasma [33] and CSF of the same MA population. **B** Heatmap of lipids (n = 10) altered in both plasma and CSF. The concentrations were auto-scaled and log-transformed for visualization. The color-scale for plasma samples differentiates values as high (yellow) and low (blue), whereas for CSF as high (pink) and low (green). HD, healthy donors; CTRL, controls subjects with unrelated cerebrovascular diseases. Statistical significance is evaluated by comparing ranks with Mann–Whitney U test. *P* values are schematized as follows: ** < 0.01; *** < 0.001

We finally searched for simultaneously altered lipids in plasma and CSF. The discriminant analysis returned n = 175 (plasma) and n = 70 (CSF) lipids respectively, and n = 10 lipids -belonging to the class of PC- resulted as commonly modulated species in plasma and CSF (Fig. 5A). The concentration of these lipid species was decreased in plasma and augmented in CSF (Fig. 5B), except for PC 42:7 that displayed an opposite trend. Thus, the previously reported lipid depletion in plasma was now counterbalanced by a CSF lipid enrichment. Such evidence seems to be of great relevance, as it was possible to identify PC as the unique class responsible for this peculiar finding. Our findings are in agreement with those previously reported by an untargeted metabolomic approach that identified a distinct LPC decrease in serum samples from MA patients, when compared to controls [27].

### Limitations

The main critical limitation of our study is the small sample size. Indeed, our pilot study focused only on a limited subset of MA patient’s samples (Caucasian, females, mean age of 47 years, bilateral MA presentation) in whom it was possible to collect CSF, because they underwent neurosurgical revascularization procedures. However, such a selected panel precisely represented the whole MA population in Western countries, which is a relevant aspect considering the rarity of the disease. Accordingly, the control subjects were numerically limited because the large part of patients suffering cerebrovascular diseases do not require neurosurgical intervention allowing CSF sampling.

In addition, we have collected data at only one time-point, after the neurological insult that determined this therapeutic option of utmost importance, thus the lack of longitudinal data prevented us from providing information on how lipid levels change over time. It is therefore rather premature to establish correlations between MA patient’s lipidomic profiles and their clinical/demographical characteristics (e.g., disease severity, as assessed by neuroimaging and Suzuki grading), given the limited case history. However, since the disease affects many of our patients bilaterally, they will undergo a double neurosurgical operation months/years apart, and this will allow us to carry out this type of investigation in the future. Nevertheless, we are gradually completing an exhaustive and up-to-date clinical database, including information on disease follow-up that will be useful, in the next future, to correlate our results with the disease status/progression, with the final goal of building a framework to guide therapeutic and preventive approaches for MA management. Most certainly, the implications of our findings are worth considering to be extended to a more numerous cohort.

## Conclusions

**Fig. 6:**
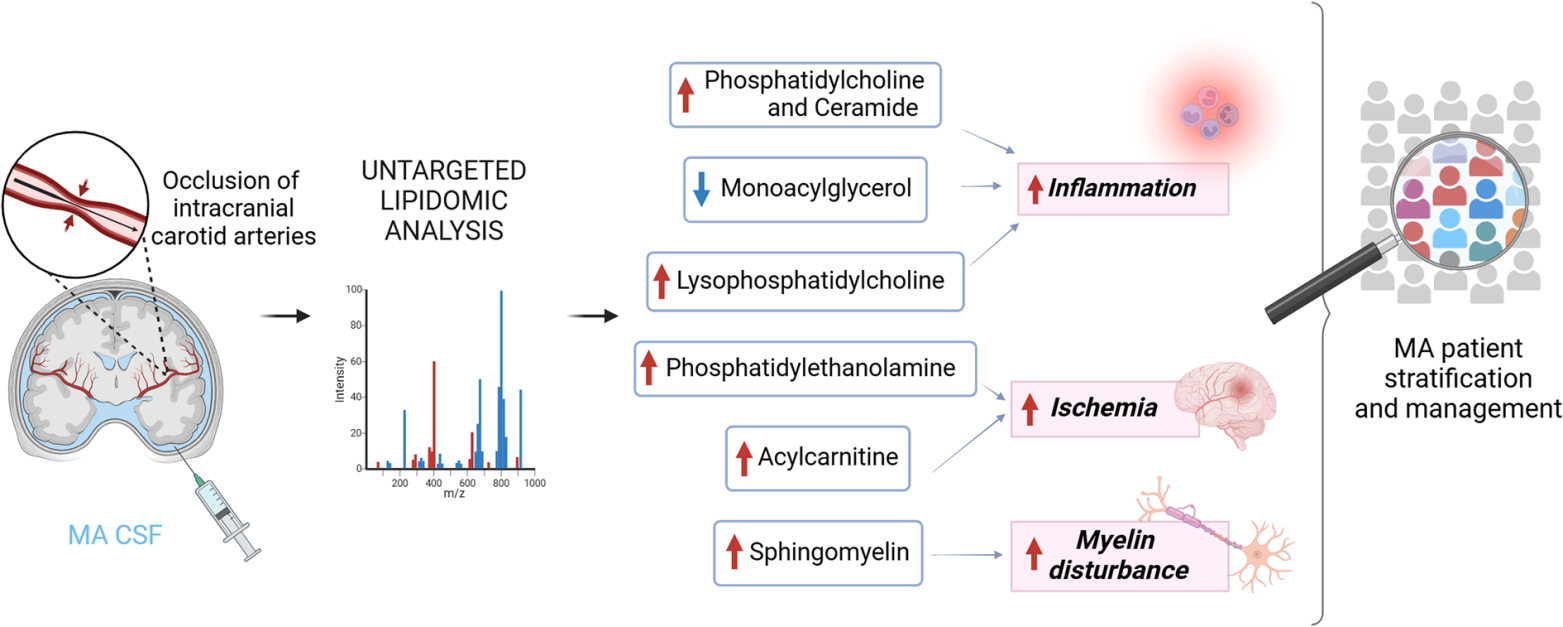
MA CSF lipidome profile analysis. The main deregulated lipid classes/species, associated with distinct pathological traits, are showed. Red arrows indicate the detection of increasing levels while the blue one stands for decreasing levels as compared to CSF of control subjects. CSF, cerebrospinal fluid; MA, moyamoya angiopathy (Figure created with BioRender)

There is an urgent need for translational research on circulating biomarkers for MA patients’ stratification, to drive personalized therapies and provide prognostic/progression advice to patients and relatives. Thus, the determination of the link between a specific measurable biomarker and significant clinical endpoints is mandatory. By capturing the intricate and multifaceted landscape of the lipidome and tracking changes in circulating lipid levels, lipidomics can depict distinct molecular phenotypes indicative of the physiological and pathological states of an organism [71, 73–76]. Based on our knowledge, this study represents the first attempt to investigate the lipidome profile of CSF samples from a small subgroup of MA patients. Indeed, this study reveals that MA CSF lipid asset is homogeneous per se, but distinctly different from that of control subjects. Interestingly, the category of phospholipids showed a significant increase in MA CSF. Phospholipids are key players in cellular phenotyping, signaling, and homeostasis, maintaining a balance between structural integrity and dynamic responsiveness [77]. Modified phospholipids can change the properties of membranes, and modulate cellular responses, or participate in signaling pathways regulating inflammation and immune response, recently suggested as second hits to trigger MA onset [4, 7]. Specifically, PC emerged as the unique class implicated in the opposite trend between plasma and CSF of the same MA cohort. Thereby, abnormally high- (in CSF) and low- (in plasma) concentrations of this lipid class may play a critical role in MA onset and progression [78] (Fig. 6). Future efforts would be focused on validating these biomarkers using a larger sample size and a targeted lipidomic approach. We will extend the correlation betweeen a panel of selected circulating biomarkers (proteins and lipids) and disease outcome, with the final goal of building a framework to guide therapeutic and preventive approaches for MA management.

## Supplementary Information

Supplementary Material 1.

## Data Availability

The study raw data supporting lipidomic reported results can be found in the MetaboLights public repository (Study-ID MTBLS9744; https://www.ebi.ac.uk/metabolights/editor/www.ebi.ac.uk/metabolights/MTBLS9744).

## Abbreviations

ACar: Acylcarnitines
AUC: Area under the curve
B: Bilateral
BBB: Blood-brain barrier
BHT: Dibutylhydroxytoluene
CE: Cholesterol esters
Cer: Ceramides
chol: Cholesterol
CHOL: Total cholesterol
CI: Confidence interval
CSF: Cerebrospinal fluid
CTRL: Controls
CVD: Cerebrovascular disease
DAG: Diacylglycerols
DHCer: Dihydroceramides
ESO: European stroke organisation
EtherPC: Ether linked phosphatidylcholines
EtherPE: Vinyl linked phosphatidylethanolamines
F: Female
Gb3: Globotriaosylceramide
GM3: Gangliosides
HDL: High-density lipoprotein
HexCer: Hexosylceramides
LacCer: Lactosylceramides
LDL: Low-density lipoprotein
LPC: Lysophosphatidylcholines
LPE: Lysophosphatidylethanolamines
MA: Moyamoya angiopathy
MAG: Monoacylglycerols
MAGL: MAG-lipase
MCA: Middle cerebral artery
mRS: Modified Rankin scale
N: Number of subjects
na: Not available
NIHSS: National institutes of health stroke scale
ns: Not significant
PC: Phosphatidylcholines
PC1: Principal component 1
PE: Phosphatidylethanolamines
PI: Phosphatidylinositoles
PLS-DA: Partial least squares discriminant analysis
RNF213: Ring finger protein 213
ROC: Receiver operating characteristic
SM: Sphingomyelins
STA: Superficial temporal artery
SULF: Sulfatides
TAG: Triacylglycerols
TG: Triglycerides
TIA: Transient ischemic attack
U: Unilateral
VIP: Variance importance in projection
y: Years

## References

[1] Shang S, Zhou D, Ya J, Li S, Yang Q, Ding Y, et al. Progress in moyamoya disease. Neurosurg Rev. 2020;43(2):371–82. 10.1007/s10143-018-0994-5. 29911252 10.1007/s10143-018-0994-5

[2] Guey S, Tournier-Lasserve E, Hervé D, Kossorotoff M. Moyamoya disease and syndromes: from genetics to clinical management. Appl Clin Genet. 2015;16(8):49–68. 10.2147/TACG.S42772 PMC4337618 25733922

[3] Kobayashi E, Saeki N, Oishi H, Hirai S, Yamaura A. Long-term natural history of hemorrhagic moyamoya disease in 42 patients. J Neurosurg. 2000;93(6):976–80. 10.3171/jns.2000.93.6.0976. 11117870 10.3171/jns.2000.93.6.0976

[4] Mejia-Munne JC, Ellis JA, Feldstein NA, Meyers PM, Connolly ES. Moyamoya and inflammation. World Neurosurg. 2017;100:575–8. 10.1016/j.wneu.2017.01.012. 28093343 10.1016/j.wneu.2017.01.012

[5] Mikami T, Suzuki H, Komatsu K, Mikuni N. Influence of inflammatory disease on the pathophysiology of moyamoya disease and quasi-moyamoya disease. Neurol Med Chir (Tokyo). 2019;59(10):361–70. 10.2176/nmc.ra.2019-0059. 31281171 10.2176/nmc.ra.2019-0059 PMC6796064

[6] Dorschel KB, Wanebo JE. Physiological and pathophysiological mechanisms of the molecular and cellular biology of angiogenesis and inflammation in moyamoya angiopathy and related vascular diseases. Front Neurol. 2023;16(14):661611. 10.3389/fneur.2023.661611. 10.3389/fneur.2023.661611 PMC10236939 37273690

[7] Singh R, Bauman MMJ, Seas A, Harrison DJ, Pennington Z, Brown NJ, et al. Association of moyamoya vasculopathy with autoimmune disease: a systematic review and pooled analysis. Neurosurg Rev. 2023;46(1):220. 10.1007/s10143-023-02123-z. 37658996 10.1007/s10143-023-02123-z

[8] Asselman C, Hemelsoet D, Eggermont D, Dermaut B, Impens F. Moyamoya disease emerging as an immune-related angiopathy. Trends Mol Med. 2022;28(11):939–50. 10.1016/j.molmed.2022.08.009. 36115805 10.1016/j.molmed.2022.08.009

[9] Sigdel TK, Shoemaker LD, Chen R, Li L, Butte AJ, Sarwal MM, et al. Immune response profiling identifies autoantibodies specific to moyamoya patients. Orphanet J Rare Dis. 2013;8:45. 10.1186/1750-1172-8-45. 23518061 10.1186/1750-1172-8-45 PMC3648437

[10] Santoro JD, Lee S, Wang AC, Ho E, Nagesh D, Khoshnood M, et al. Increased autoimmunity in individuals with down syndrome and moyamoya disease. Front Neurol. 2021;8(12):724969. 10.3389/fneur.2021.724969. 10.3389/fneur.2021.724969 PMC8455812 34566869

[11] Lanterna LA, Galliani S, Zangari R, Conti L, Brembilla C, Gritti P, et al. Thyroid autoantibodies and the clinical presentation of moyamoya disease: a prospective study. J Stroke Cerebrovasc Dis. 2018;27:1194–9. 10.1016/j.jstrokecerebrovasdis.2017.11.037. 29305275 10.1016/j.jstrokecerebrovasdis.2017.11.037

[12] Sum CH, Tsang ACO, Cheng KK, Ho WW, Leung GKK, Lui WM. Surgical revascularization for moyamoya angiopathy: clinical and radiological outcomes of direct and indirect bypasses in 86 affected hemispheres. J Clin Neurosci. 2022;99:66–72. 10.1016/j.jocn.2022.02.036. 35255359 10.1016/j.jocn.2022.02.036

[13] Young AM, Karri SK, Ogilvy CS, Zhao N. Is there a role for treating inflammation in moyamoya disease? A review of histopathology, genetics, and signaling cascades. Front Neurol. 2013;14(4):105. 10.3389/fneur.2013.00105 PMC3742998 23966972

[14] Guey S, Kraemer M, Hervé D, Ludwig T, Kossorotoff M, Bergametti F, et al. FREX consortium. Rare RNF213 variants in the C-terminal region encompassing the RING-finger domain are associated with moyamoya angiopathy in Caucasians. Eur J Hum Genet. 2017;25(8):995–1003. 10.1038/ejhg.2017.92. 28635953 10.1038/ejhg.2017.92 PMC5567158

[15] Zhang H, Zheng L, Feng L. Epidemiology, diagnosis and treatment of moyamoya disease. Exp Ther Med. 2019;17(3):1977–84. 10.3892/etm.2019.7198. 30867689 10.3892/etm.2019.7198 PMC6395994

[16] Baba T, Houkin K, Kuroda S. Novel epidemiological features of moyamoya disease. J Neurol Neurosurg Psychiatry. 2008;79(8):900–4. 10.1136/jnnp.2007.130666. 18077479 10.1136/jnnp.2007.130666

[17] Kamada F, Aoki Y, Narisawa A, Abe Y, Komatsuzaki S, Kikuchi A, et al. A genome-wide association study identifies RNF213 as the first moyamoya disease gene. J Hum Genet. 2011;56(1):34–40. 10.1038/jhg.2010.132. 21048783 10.1038/jhg.2010.132

[18] Cao L, Dong Y, Sun K, Li D, Wang H, Li H, et al. Experimental animal models for moyamoya disease: a species-oriented scoping review. Front Surg. 2022;1(9):929871. 10.3389/fsurg.2022.929871. 10.3389/fsurg.2022.929871 PMC9283787 35846951

[19] Correia M, Silva I, Gabriel D, Simrén J, Carneiro A, Ribeiro S, et al. Early plasma biomarker dynamic profiles are associated with acute ischemic stroke outcomes. Eur J Neurol. 2022;29(6):1630–42. 10.1111/ene.15273. 35124870 10.1111/ene.15273

[20] Michaëlsson I, Hallén T, Carstam L, Laesser M, Björkman-Burtscher IM, Sörbo A, et al. Circulating brain injury biomarkers: a novel method for quantification of the impact on the brain after tumor surgery. Neurosurgery. 2023;93(4):847–56. 10.1227/neu.0000000000002510. 37140203 10.1227/neu.0000000000002510 PMC10637403

[21] Zeng Y, Yue H, Cao B, Li Y, Yang M, Mao C. Target-triggered formation of artificial enzymes on filamentous phage for ultrasensitive direct detection of circulating mirna biomarkers in clinical samples. Angew Chem Int Ed Engl. 2022;61(45):e202210121. 10.1002/anie.202210121. 36108201 10.1002/anie.202210121

[22] Gatti L, Bersano A, Gorla G, Pollaci G, Carrozzini T, Potenza A. Multi-omic approaches for biomarker discovery in moyamoya disease. Ann Clin Transl Neurol. 2024;4(2):e270. 10.1002/ctd2.270.

[23] Carrozzini T, Pollaci G, Gorla G, Potenza A, Rifino N, Acerbi F, et al. Proteome profiling of the dura mater in patients with moyamoya angiopathy. Int J Mol Sci. 2023;24(13):11194. 10.3390/ijms241311194. 37446373 10.3390/ijms241311194 PMC10342562

[24] Wang Z, Ji C, Han Q, Wang Z, Huang Y. Data-independent acquisition-based serum proteomic profiling of adult moyamoya disease patients reveals the potential pathogenesis of vascular changes. J Mol Neurosci. 2022;72(12):2473–85. 10.1007/s12031-022-02092-w. 36520382 10.1007/s12031-022-02092-w

[25] Wang X, Han C, Jia Y, Wang J, Ge W, Duan L. Proteomic profiling of exosomes from hemorrhagic moyamoya disease and dysfunction of mitochondria in endothelial cells. Stroke. 2021;52(10):3351–61. 10.1161/STROKEAHA.120.032297. 34334053 10.1161/STROKEAHA.120.032297

[26] Yu J, Chen T, Li X, Chen J, Wei W, Zhang J. Liquid chromatography coupled to mass spectrometry metabolomic analysis of cerebrospinal fluid revealed the metabolic characteristics of moyamoya disease. Front Neurol. 2024;15(15):1298385. 10.3389/fneur.2024.1298385. 38426176 10.3389/fneur.2024.1298385 PMC10902010

[27] He S, Wang Y, Liu Z, Zhang J, Hao X, Wang X, et al. Metabolomic signatures associated with pathological angiogenesis in moyamoya disease. Clin Transl Med. 2023;13(12):e1492. 10.1002/ctm2.1492. 38037492 10.1002/ctm2.1492 PMC10689969

[28] Pollaci G, Gorla G, Potenza A, Carrozzini T, Canavero I, Bersano A, et al. Novel multifaceted roles for RNF213 protein. Int J Mol Sci. 2022;23(9):4492. 10.3390/ijms23094492. 35562882 10.3390/ijms23094492 PMC9099590

[29] Fantini J, Barrantes FJ. Sphingolipid/cholesterol regulation of neurotransmitter receptor conformation and function. Biochim Biophys Acta. 2009;1788(11):2345–61. 10.1016/j.bbamem.2009.08.016. 19733149 10.1016/j.bbamem.2009.08.016

[30] Zhang J, Liu Q. Cholesterol metabolism and homeostasis in the brain. Protein Cell. 2015;6(4):254–64. 10.1007/s13238-014-0131-3. 25682154 10.1007/s13238-014-0131-3 PMC4383754

[31] He K, Wang X, Gu Y, Tong X, Qin X, Liao Y, et al. In-depth cerebrovascular lipidomics profiling for discovering novel biomarkers and mechanisms in moyamoya and intracranial atherosclerotic disease. Int J Surg. 2024;111(1):1607–13. 10.1097/JS9.0000000000002092. 10.1097/JS9.0000000000002092 PMC11745776 39352103

[32] Gorla G, Potenza A, Carrozzini T, Pollaci G, Acerbi F, Vetrano IG, et al. Angiopoietin-2 associates with poor prognosis in moyamoya angiopathy. Ann Clin Transl Neurol. 2024;11(6):1590–603. 10.1002/acn3.52076. 38655722 10.1002/acn3.52076 PMC11187837

[33] Dei Cas M, Carrozzini T, Pollaci G, Potenza A, Nava S, Canavero I, et al. Plasma lipid profiling contributes to untangle the complexity of moyamoya arteriopathy. Int J Mol Sci. 2021;22(24):13410. 10.3390/ijms222413410. 34948203 10.3390/ijms222413410 PMC8708587

[34] Research Committee on the Pathology and Treatment of Spontaneous Occlusion of the Circle of Willis; Health Labour Sciences Research Grant for Research on Measures for Infractable Diseases. Guidelines for diagnosis and treatment of moyamoya disease (spontaneous occlusion of the circle of Willis). Neurol Med Chir (Tokyo). 2012;52(5):245–66. 10.2176/nmc.52.245 10.2176/nmc.52.245 22870528

[35] Bersano A, Bedini G, Nava S, Acerbi F, Sebastiano DR, Binelli S, et al.; GEN-O-MA study group. GEN-O-MA project: an Italian network studying clinical course and pathogenic pathways of moyamoya disease-study protocol and preliminary results. Neurol Sci. 2019;40(3):561–570. 10.1007/s10072-018-3664-z. 10.1007/s10072-018-3664-z 30604336

[36] Bersano A, Khan N, Fuentes B, Acerbi F, Canavero I, Tournier-Lasserve E, et al. European stroke organisation (ESO) guidelines on moyamoya angiopathy endorsed by vascular European reference network (VASCERN). Eur Stroke J. 2023;8(1):55–84. 10.1177/23969873221144089. 37021176 10.1177/23969873221144089 PMC10069176

[37] Suzuki J, Takaku A. Cerebrovascular, “moyamoya” disease. Disease showing abnormal net-like vessels in base of brain. Arch Neurol. 1969;20(3):288–99. 10.1001/archneur.1969.00480090076012. 5775283 10.1001/archneur.1969.00480090076012

[38] MS-DIAL ver. 4.0. RIKEN Center for Sustainable Resource Science, Suehiro-cho, Tsurumi-ku, Yokohama City, Kanagawa, Japan. http://prime.psc.riken.jp/compms /msdial/main.html. Accessed 29 Dec 2023.

[39] MetaboLights Database. EMBL-EBI, Wellcome Genome Campus, Hinxton, Cambridgeshire, UK. https://www.ebi.ac.uk/metabolights/editor/study/MTBLS9744/descriptors. Accessed 15 May 2024

[40] MetaboAnalyst ver. 5.0. McGill Data Center and Compute Canada, Montréal, Canada. https://www.metaboanalyst.ca. Accessed 17 Dec 2023

[41] Tinelli F, Nava S, Arioli F, Bedini G, Scelzo E, Lisini D, et al. Vascular remodeling in moyamoya angiopathy: from peripheral blood mononuclear cells to endothelial cells. Int J Mol Sci. 2020;21(16):5763. 10.3390/ijms21165763. 32796702 10.3390/ijms21165763 PMC7460840

[42] Canavero I, Vetrano IG, Zedde M, Pascarella R, Acerbi F, et al. Clinical management of moyamoya patients. J Clin Med. 2021;10(16):3628. 10.3390/jcm10163628. 34441923 10.3390/jcm10163628 PMC8397113

[43] Yuan H, Zhu B, Li C, Zhao Z. Ceramide in cerebrovascular diseases. Front Cell Neurosci. 2023;2(17):1191609. 10.3389/fncel.2023.1191609. 10.3389/fncel.2023.1191609 PMC10272456 37333888

[44] Adibhatla RM, Hatcher JF. Cytidine 5’-diphosphocholine (CDP-choline) in stroke and other CNS disorders. Neurochem Res. 2005;30(1):15–23. 10.1007/s11064-004-9681-8. 15756928 10.1007/s11064-004-9681-8 PMC1934404

[45] Magaquian D, Delgado Ocaña S, Perez C, Banchio C. Phosphatidylcholine restores neuronal plasticity of neural stem cells under inflammatory stress. Sci Rep. 2021;11(1):22891. 10.1038/s41598-021-02361-5. 34819604 10.1038/s41598-021-02361-5 PMC8613233

[46] Blondeau N, Lauritzen I, Widmann C, Lazdunski M, Heurteaux C. A potent protective role of lysophospholipids against global cerebral ischemia and glutamate excitotoxicity in neuronal cultures. J Cereb Blood Flow Metab. 2002;22(7):821–34. 10.1097/00004647-200207000-00007. 12142567 10.1097/00004647-200207000-00007

[47] Huang M, Xu S, Zhou M, Luo J, Zha F, Shan L, et al. Lysophosphatidylcholines and phosphatidylcholines as biomarkers for stroke recovery. Front Neurol. 2022;15(13):1047101. 10.3389/fneur.2022.1047101. 10.3389/fneur.2022.1047101 PMC9797831 36588912

[48] Choi JY, Kim JS, Kim JH, Oh K, Koh SB, Seo WK. High free fatty acid level is associated with recurrent stroke in cardioembolic stroke patients. Neurology. 2014;82(13):1142–8. 10.1212/WNL.0000000000000264. 24587477 10.1212/WNL.0000000000000264

[49] Seo WK, Jo G, Shin MJ, Oh K. Medium-chain acylcarnitines are associated with cardioembolic stroke and stroke recurrence. Arterioscler Thromb Vasc Biol. 2018;38(9):2245–53. 10.1161/ATVBAHA.118.311373. 30026276 10.1161/ATVBAHA.118.311373

[50] Au A. Metabolomics and lipidomics of ischemic stroke. Adv Clin Chem. 2018;85:31–69. 10.1016/bs.acc.2018.02.002. 29655461 10.1016/bs.acc.2018.02.002

[51] Sun R, Li Y, Cai M, Cao Y, Piao X. Discovery of a new biomarker pattern for differential diagnosis of acute ischemic stroke using targeted metabolomics. Front Neurol. 2019;19(10):1011. 10.3389/fneur.2019.01011. 10.3389/fneur.2019.01011 PMC6761218 31608005

[52] Guasch-Ferré M, Zheng Y, Ruiz-Canela M, Hruby A, Martínez-González MA, Clish CB, et al. Plasma acylcarnitines and risk of cardiovascular disease: effect of Mediterranean diet interventions. Am J Clin Nutr. 2016;103(6):1408–16. 10.3945/ajcn.116.130492. 27099249 10.3945/ajcn.116.130492 PMC4881000

[53] Meikle PJ, Wong G, Tsorotes D, Barlow CK, Weir JM, Christopher MJ, MacIntosh GL, Goudey B, Stern L, Kowalczyk A, Haviv I, White AJ, Dart AM, Duffy SJ, Jennings GL, Kingwell BA. Plasma lipidomic analysis of stable and unstable coronary artery disease. Arterioscler Thromb Vasc Biol. 2011;31(11):2723–32. 10.1161/ATVBAHA.111.234096. 21903946 10.1161/ATVBAHA.111.234096

[54] Petkevicius K, Virtue S, Bidault G, Jenkins B, Çubuk C, Morgantini C, et al. Accelerated phosphatidylcholine turnover in macrophages promotes adipose tissue inflammation in obesity. Elife. 2019;16(8):e47990. 10.7554/eLife.47990. 10.7554/eLife.47990 PMC6748830 31418690

[55] Liu P, Zhu W, Chen C, Yan B, Zhu L, Chen X, Peng C. The mechanisms of lysophosphatidylcholine in the development of diseases. Life Sci. 2020;15(247):117443. 10.1016/j.lfs.2020.117443. 10.1016/j.lfs.2020.117443 32084434

[56] Czubowicz K, Jęśko H, Wencel P, Lukiw WJ, Strosznajder RP. The role of ceramide and sphingosine-1-phosphate in Alzheimer’s disease and other neurodegenerative disorders. Mol Neurobiol. 2019;56(8):5455. 10.1007/s12035-018-1448-3. 10.1007/s12035-018-1448-3 PMC6614129 30612333

[57] Kang SC, Kim BR, Lee SY, Park TS. Sphingolipid metabolism and obesity-induced inflammation. Front Endocrinol (Lausanne). 2013;4(4):67. 10.3389/fendo.2013.00067. 23761785 10.3389/fendo.2013.00067 PMC3671289

[58] Capodivento G, De Michelis C, Carpo M, Fancellu R, Schirinzi E, Severi D, et al. CSF sphingomyelin: a new biomarker of demyelination in the diagnosis and management of CIDP and GBS. J Neurol Neurosurg Psychiatry. 2021;92(3):303–10. 10.1136/jnnp-2020-324445. 33093191 10.1136/jnnp-2020-324445 PMC7892388

[59] Hara S, Hori M, Hagiwara A, Tsurushima Y, Tanaka Y, Maehara T, et al. Myelin and axonal damage in normal-appearing white matter in patients with moyamoya disease. AJNR Am J Neuroradiol. 2020;41(9):1618–24. 10.3174/ajnr.A6708. 32855183 10.3174/ajnr.A6708 PMC7583130

[60] Ghidoni R, Caretti A, Signorelli P. Role of sphingolipids in the pathobiology of lung inflammation. Mediators Inflamm. 2015;2015:487508. 10.1155/2015/487508. 26770018 10.1155/2015/487508 PMC4681829

[61] Gulati S, Liu Y, Munkacsi AB, Wilcox L, Sturley SL. Sterols and sphingolipids: dynamic duo or partners in crime? Prog Lipid Res. 2010;49(4):353–65. 10.1016/j.plipres.2010.03.003. 20362613 10.1016/j.plipres.2010.03.003 PMC2938828

[62] Chang CC, Lee CY, Chang ET, Cruz JC, Levesque MC, Chang TY. Recombinant acyl-CoA:cholesterol acyltransferase-1 (ACAT-1) purified to essential homogeneity utilizes cholesterol in mixed micelles or in vesicles in a highly cooperative manner. J Biol Chem. 1998;273(52):35132–41. 10.1074/jbc.273.52.35132. 9857049 10.1074/jbc.273.52.35132

[63] Pitkänen AS, Halonen TO, Kilpeläinen HO, Riekkinen PJ. Cholesterol esterase activity in cerebrospinal fluid of multiple sclerosis patients. J Neurol Sci. 1986;74(1):45–53. 10.1016/0022-510x(86)90190-5. 3723135 10.1016/0022-510x(86)90190-5

[64] Cutler RG, Pedersen WA, Camandola S, Rothstein JD, Mattson MP. Evidence that accumulation of ceramides and cholesterol esters mediates oxidative stress-induced death of motor neurons in amyotrophic lateral sclerosis. Ann Neurol. 2002;52(4):448–57. 10.1002/ana.10312. 12325074 10.1002/ana.10312

[65] Gerl MJ, Vaz WLC, Domingues N, Klose C, Surma MA, Sampaio JL, et al. Cholesterol is inefficiently converted to cholesteryl esters in the blood of cardiovascular disease patients. Sci Rep. 2018;8(1):14764. 10.1038/s41598-018-33116-4. 30282999 10.1038/s41598-018-33116-4 PMC6170447

[66] Brennan E, Kantharidis P, Cooper ME, Godson C. Pro-resolving lipid mediators: regulators of inflammation, metabolism and kidney function. Nat Rev Nephrol. 2021;17(11):725–39. 10.1038/s41581-021-00454-y. 34282342 10.1038/s41581-021-00454-y PMC8287849

[67] Habib A, Chokr D, Wan J, Hegde P, Mabire M, Siebert M, et al. Inhibition of monoacylglycerol lipase, an anti-inflammatory and antifibrogenic strategy in the liver. Gut. 2019;68(3):522–32. 10.1136/gutjnl-2018-316137. 30301768 10.1136/gutjnl-2018-316137

[68] Dinh TP, Carpenter D, Leslie FM, Freund TF, Katona I, Sensi SL, et al. Brain monoglyceride lipase participating in endocannabinoid inactivation. Proc Natl Acad Sci U S A. 2002;99(16):10819–24. 10.1073/pnas.152334899. 12136125 10.1073/pnas.152334899 PMC125056

[69] Nomura DK, Morrison BE, Blankman JL, Long JZ, Kinsey SG, Marcondes MC, et al. Endocannabinoid hydrolysis generates brain prostaglandins that promote neuroinflammation. Science. 2011;334(6057):809–13. 10.1126/science.1209200. 22021672 10.1126/science.1209200 PMC3249428

[70] Cao Y, Wang W, Xu Y, Yang B, Wang Y. Enzymatic synthesis of extremely pure triacylglycerols enriched in conjugated linoleic acids. Molecules. 2013;18(8):9704–16. 10.3390/molecules18089704. 23945644 10.3390/molecules18089704 PMC6270589

[71] Camerer E. Endotoxemia rocks sphingolipid metabolism at the blood-brain barrier: an Editorial Highlight for ‘Alteration of sphingolipid metabolism as a putative mechanism underlying LPS-induced BBB disruption’ on page 172. J Neurochem. 2018;144(2):115–7. 10.1111/jnc.14246. 29285771 10.1111/jnc.14246

[72] Narducci A, Yasuyuki K, Onken J, Blecharz K, Vajkoczy P. In vivo demonstration of blood-brain barrier impairment in moyamoya disease. Acta Neurochir (Wien). 2019;161(2):371–8. 10.1007/s00701-019-03811-w. 30675657 10.1007/s00701-019-03811-w

[73] van Meer G, Voelker DR, Feigenson GW. Membrane lipids: where they are and how they behave. Nat Rev Mol Cell Biol. 2008;9(2):112–24. 10.1038/nrm2330. 18216768 10.1038/nrm2330 PMC2642958

[74] Chiurchiù V, Leuti A, Maccarrone M. Bioactive lipids and chronic inflammation: managing the fire within. Front Immunol. 2018;29(9):38. 10.3389/fimmu.2018.00038. 10.3389/fimmu.2018.00038 PMC5797284 29434586

[75] Hussain G, Wang J, Rasul A, Anwar H, Imran A, Qasim M, et al. Role of cholesterol and sphingolipids in brain development and neurological diseases. Lipids Health Dis. 2019;18(1):26. 10.1186/s12944-019-0965-z. 30683111 10.1186/s12944-019-0965-z PMC6347843

[76] van Kruining D, Luo Q, van Echten-Deckert G, Mielke MM, Bowman A, Ellis S, et al. Sphingolipids as prognostic biomarkers of neurodegeneration, neuroinflammation, and psychiatric diseases and their emerging role in lipidomic investigation methods. Adv Drug Deliv Rev. 2020;159:232–44. 10.1016/j.addr.2020.04.009. 32360155 10.1016/j.addr.2020.04.009 PMC7665829

[77] Santos M, Melo T, Maurício T, Ferreira H, Domingues P, Domingues R. The non-enzymatic oxidation of phosphatidylethanolamine and phosphatidylserine and their intriguing roles in inflammation dynamics and diseases. FEBS Lett. 2024;598(17):2174–89. 10.1002/1873-3468.14992. 39097985 10.1002/1873-3468.14992

[78] van der Veen JN, Kennelly JP, Wan S, Vance JE, Vance DE, Jacobs RL. The critical role of phosphatidylcholine and phosphatidylethanolamine metabolism in health and disease. Biochim Biophys Acta Biomembr. 2017;1859(9 Pt B):1558–72. 10.1016/j.bbamem.2017.04.006. 28411170 10.1016/j.bbamem.2017.04.006

