# Supplementary material for: Lipidomic profiling of the cerebrospinal fluid in moyamoya angiopathy patients
Source: Orphanet J Rare Dis. 2025 May 23;20:243. doi: 10.1186/s13023-025-03782-5 (PMC12101001; doi:10.1186/s13023-025-03782-5)
Supplement: Supplementary file 1 — Supplementary Material 1. [file 13023_2025_3782_MOESM1_ESM.pdf]

Additional file 1:

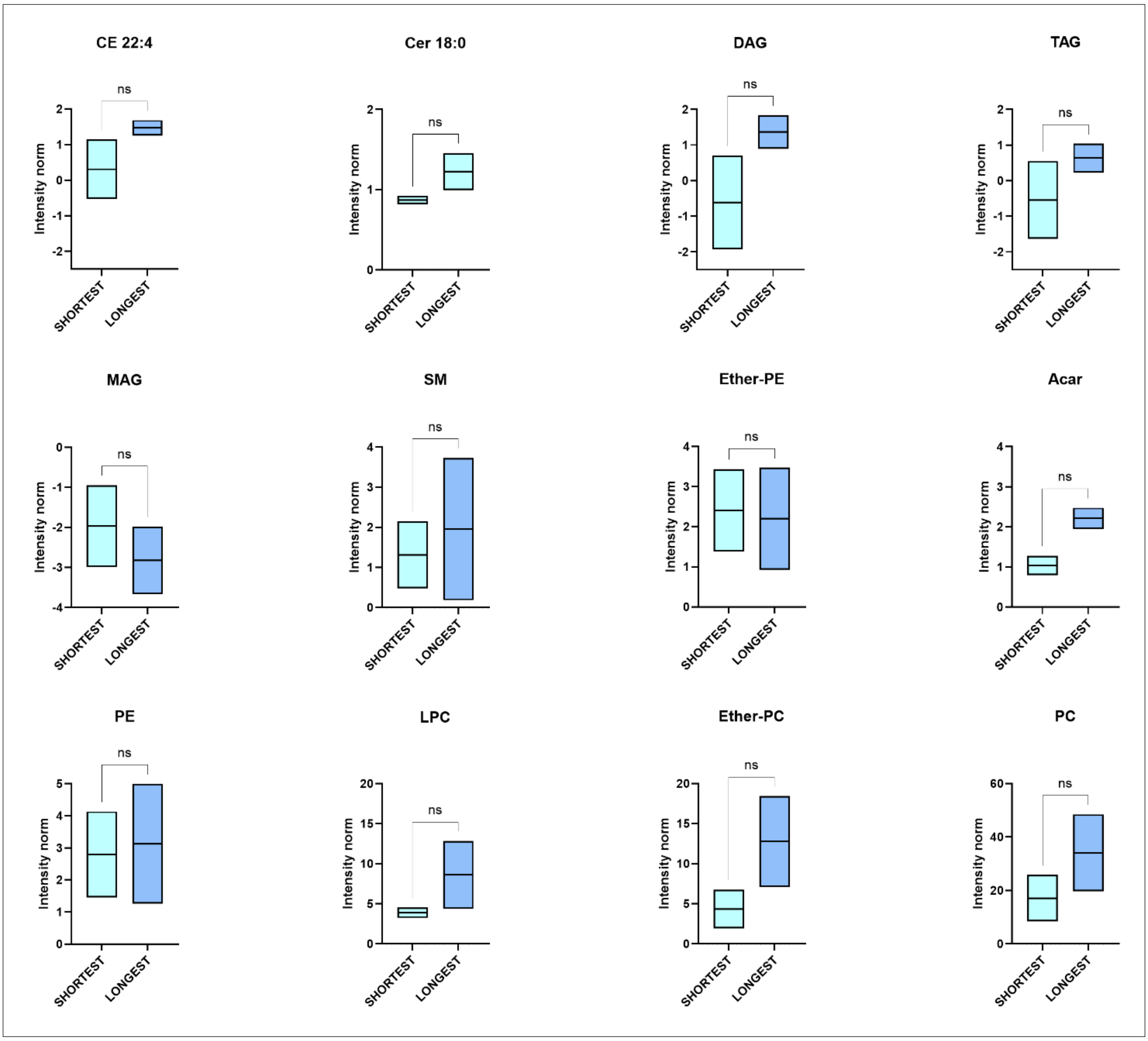

VIP lipid class concentrations in MA patients, grouped based on MA duration. The MA duration stands for the amount of months spent between index event (disease onset) and CSF sampling occurring during neovascularization procedures. The shortest MA duration refers to 5 months, while the longest one account for 37 months. Floating bars represent values (min to max). The line indicates a median value. Statistical significance is evaluated by comparing ranks with Mann-Whitney U test. Not statistically significant differences are denoted as *ns* (not significant). The nomenclature of lipid classes has been reported in Table 2.

Additional file 2:

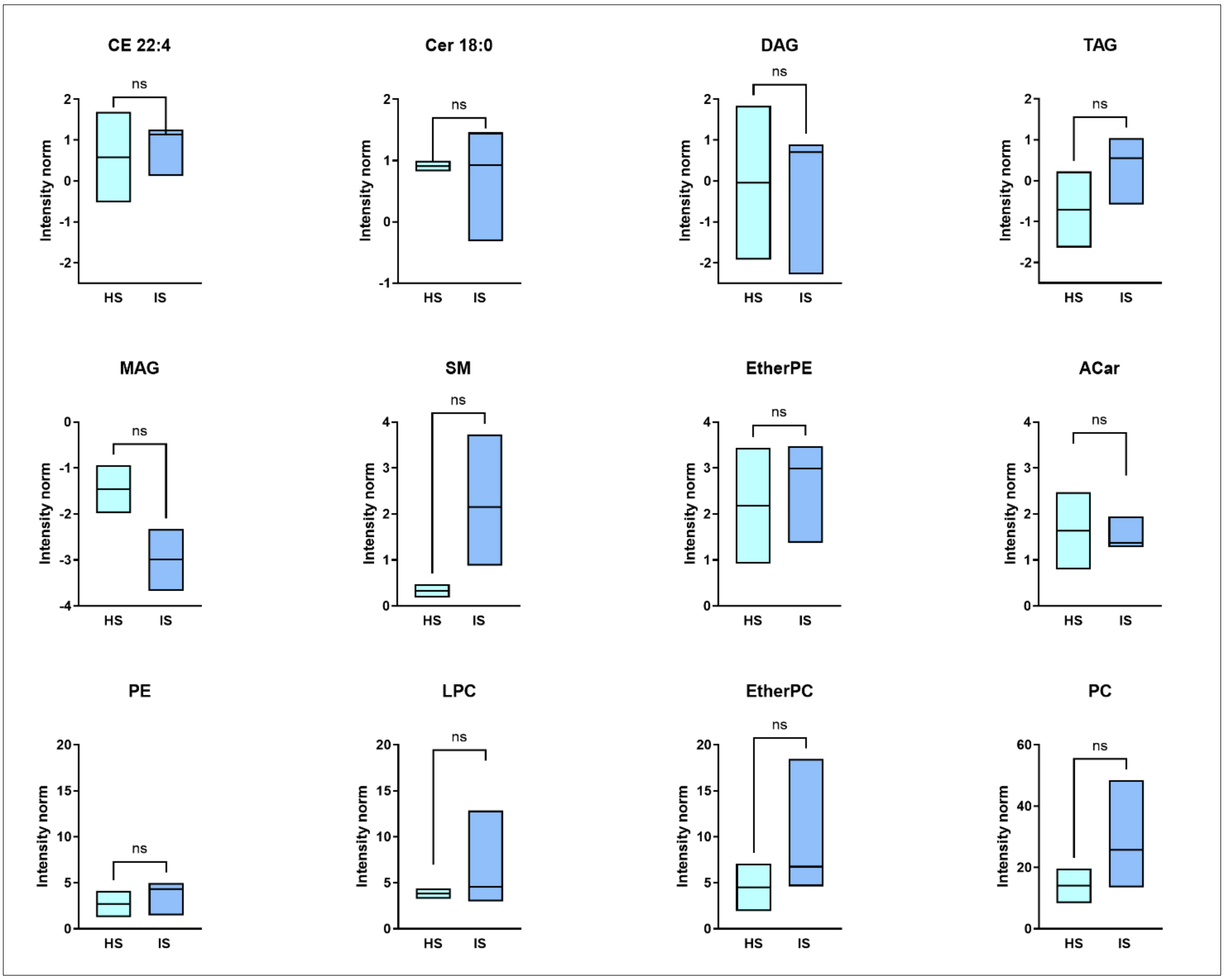

VIP lipid class concentrations in MA patients, grouped based on MA presentation. The MA cohort has been clustered, by differentiating patients with a haemorrhagic (HS) MA presentation from those with an ischemic (IS) one. Of note, the only one patient characterized by a TIA has been included within the IS group. Floating bars represent values (min to max). The line indicates a median value. Statistical significance is evaluated by comparing ranks with Mann-Whitney U test. Not statistically significant differences are denoted as *ns* (not significant). The nomenclature of lipid classes has been reported in Table 2.

Additional file 3:

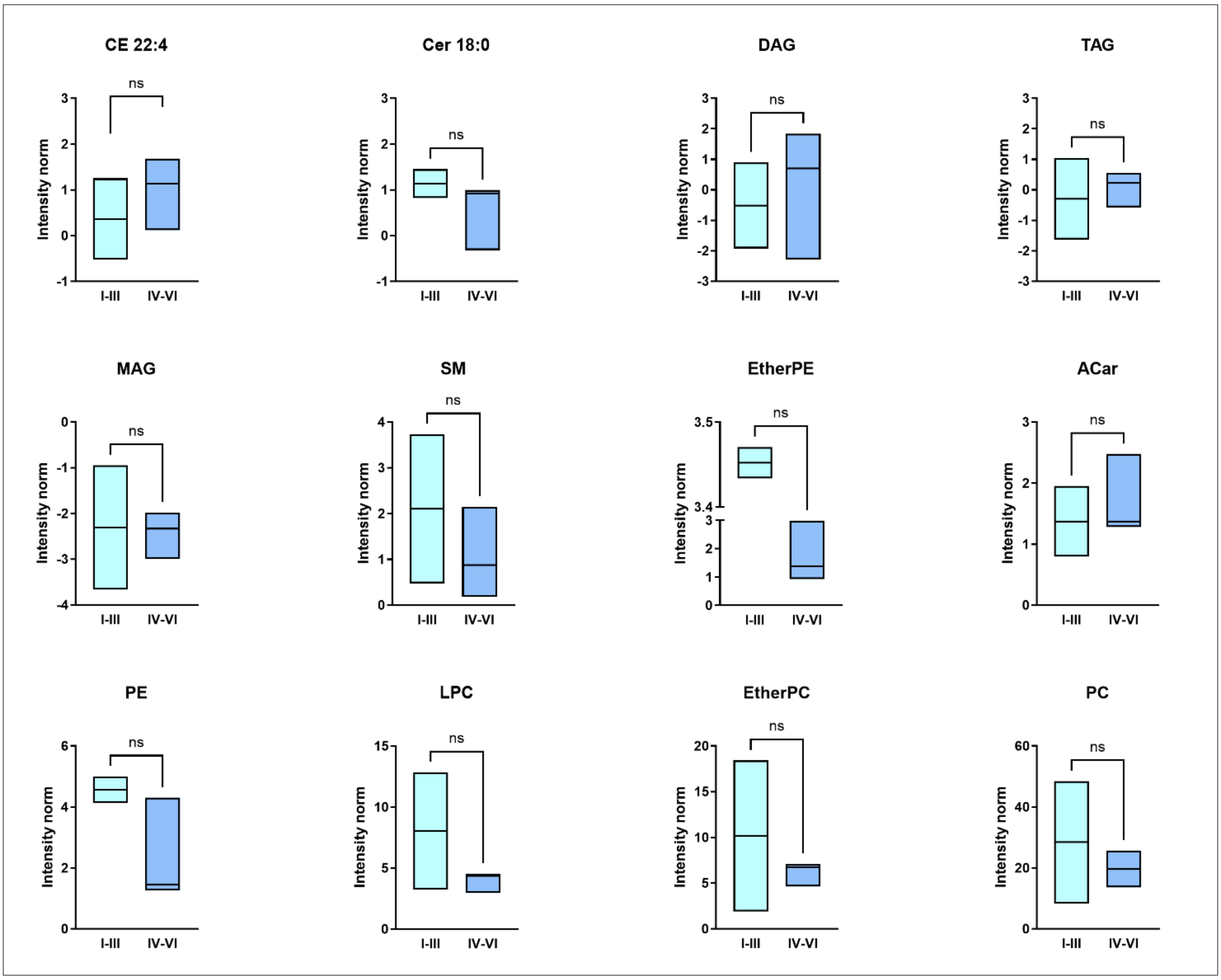

VIP lipid class concentrations in MA patients, grouped based on Suzuki grading. The MA cohort has been clustered, by differentiating patients characterized by a Suzuki grading I-III *vs* Suzuki grading IV-VI. Floating bars represent values (min to max). The line indicates a median value. Statistical significance is evaluated by comparing ranks with Mann-Whitney U test. Not statistically significant differences are denoted as *ns* (not significant). The nomenclature of lipid classes has been reported in Table 2.

Additional file 4:

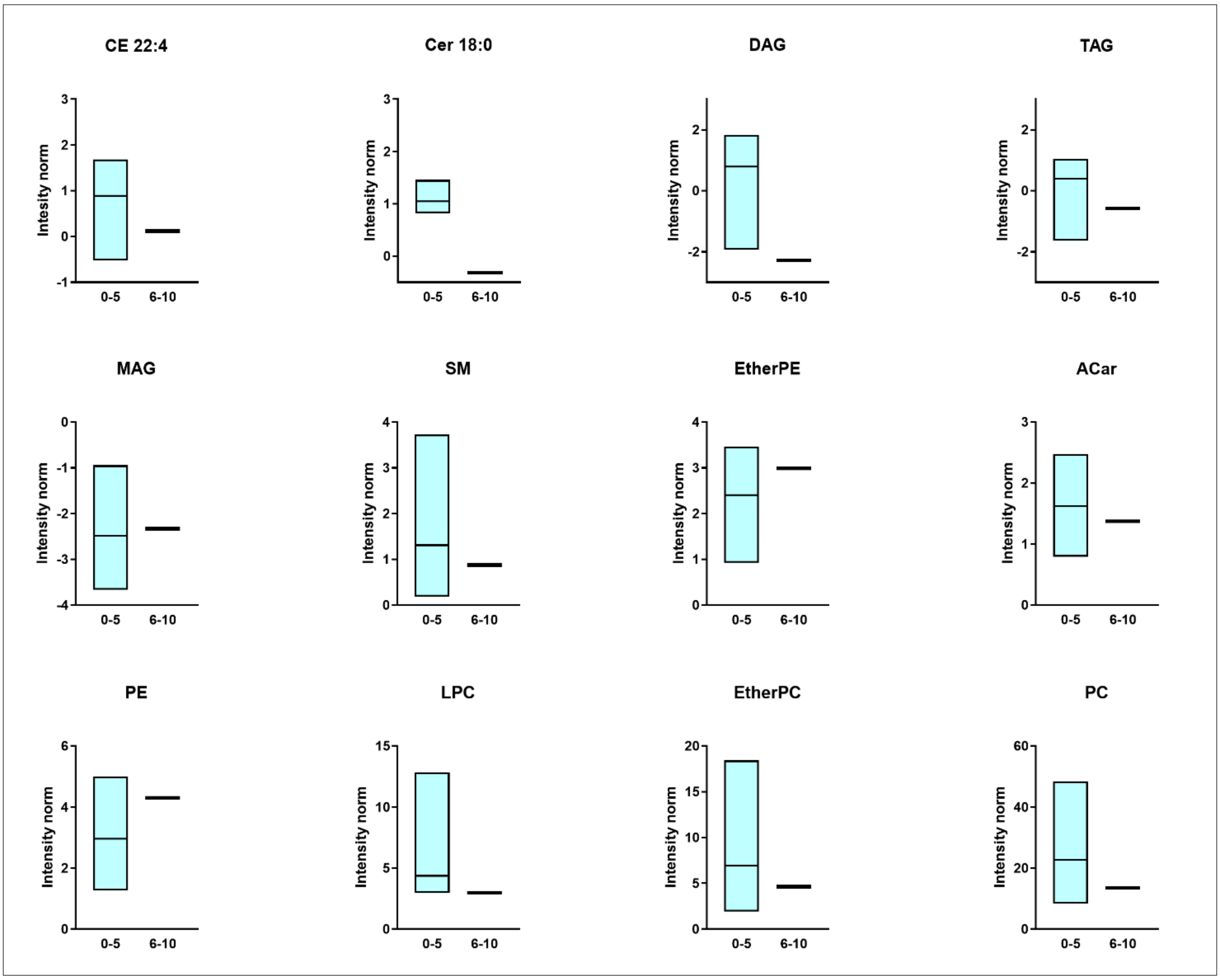

VIP lipid class concentrations in MA patients, grouped based on NIH Stroke Scale/Score (NIHSS). The MA cohort has been clustered, by differentiating patients characterized by NIHSS 0-5 vs 6-10. Floating bars represent values (min to max), as far as NIHSS 0-1 group. The line indicates a median value. Only one patient is associated to a NIHSS equal to 8 (NIHSS 6-10 group). Thus, due to the paucity of data characterizing this specific MA subgroup, it has not been possible to perform a proper statistical analysis.

Additional file 5:

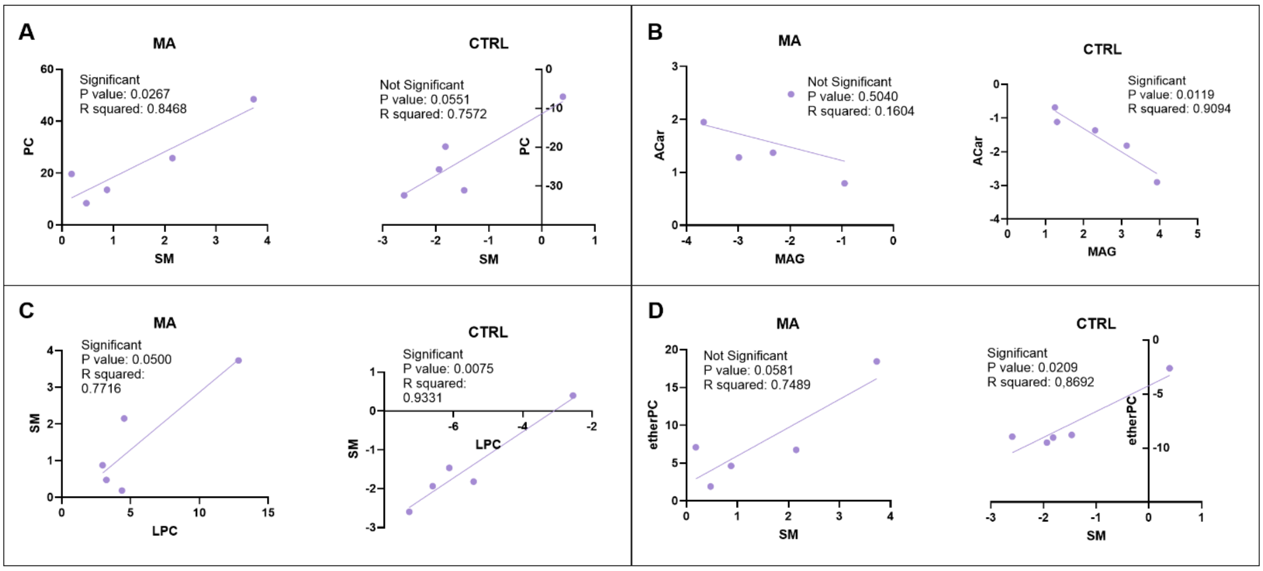

Simple linear regression model. Statistically significant simple linear regression analyses of selected lipid classes that showed VIP≥1.5 in CSF of MA patients as compared to CTRL subjects; (A) PC/SM; (B) ACar/MAG; (C) SM/LPC; (D) ether PC/SM. The nomenclature of lipid classes has been reported in Table 2.
